# What factors are associated with children being taken into care by the state after initial contact with services? A survival analysis of Children’s Social Care data in Liverpool

**DOI:** 10.1136/bmjph-2024-001130

**Published:** 2024-10-07

**Authors:** Philip McHale, Luís Filipe, Sarah Hodgson, Davara Bennett, Benjamin Barr

**Affiliations:** 1 Department of Public Health, Policy and Systems, University of Liverpool, Liverpool, UK; 2 Lancaster University, Lancaster, UK; 3 Wigan Council, Wigan, UK

**Keywords:** Public Health, Statistics and Numerical Data, Sociodemographic Factors

## Abstract

**Background:**

Increasing numbers of children in the UK are being taken into care, with adverse consequences for children and unsustainable costs for local government. It is crucial that local authorities better understand which children are most at risk to target preventative interventions.

**Objectives:**

To identify predictors of children becoming ‘looked after’ (taken into care by the state) among children known to a local authority.

**Methods:**

Secondary analysis of routinely collected Children’s Social Care data including all children who first became known to social care in Liverpool between April 2019 and March 2022, (excluding unaccompanied asylum seeker children). Outcome was time (in months) between first contact with social care and either becoming looked after or reaching the censoring date (March 2022). Survival analysis was undertaken using a discrete time hazard model.

**Results:**

5808 children under 19 became known to Children’s Social Care during the study, 377 of whom subsequently became looked after. Black and Asian children were more likely to become looked after, as were those known to social care services pre-birth or in the first year. Key risk factors that increased hazard of becoming looked after were neglect, sexual abuse, emotional abuse, drug and/or alcohol use in the household or the child and mental ill health in the household. Children who had a Child in Need intervention were less likely to become looked after. Children who had a Child Protection Plan were more likely to become looked after.

**Conclusion:**

In our study of routinely collected Children’s Social Care data, we have identified the key factors that increased the hazard of a child becoming looked after. These findings highlight potential areas for service change and can be used to inform risk prediction and preventative action, however, the local context may influence the generalisability of our findings to other settings.

**WHAT IS ALREADY KNOWN ON THIS TOPIC**

**WHAT THIS STUDY ADDS**

- Neglect, sexual abuse, emotional abuse, drug and/or alcohol use in the household or the child, mental ill health in the household and the use of a Child Protection Plan all were associated with an increased risk of a child becoming looked after. The use of a Child in Need intervention was associated with a reduced risk of a child becoming looked after. Domestic violence was not significantly associated with the risk of a child becoming looked after.

**HOW THIS STUDY MIGHT AFFECT RESEARCH, PRACTICE OR POLICY**

- Costs of Children’s Social Care are increasing and create severe burdens on local government finances; our findings can be used to identify children at risk, informing preventative action and early intervention.

## Introduction

 Children looked after (CLA) are children who are under the care of local government for between 24 and 72 hours. In the UK, a CLA intervention is a statutory tool that most often involves the removal of a child from their family home. CLA may be placed with foster parents, in a residential children’s home or other residential setting.^1^ Total numbers of CLA have been increasing consistently while new CLA plans have increased over the past 2 years. In March 2023, the most recent data for England, there were nearly 1800 more CLA than in March 2022, a 2% increase.^2 3^ There are significant inequalities seen in the increasing trend in CLA rates, with higher numbers of CLA in areas with higher rates of child poverty.^4^

In the UK there are three levels of statutory intervention for children who are believed to be at risk: Child in Need, Child Protection Plan and CLA. Each level of intervention refers to a differing level of need and support required. Child in Need intervention is a non-compulsory intervention (the support offered can be refused by the family) that aims to provide support to maximise the welfare and development of the child.^5 6^ A Child Protection Plan is a more acute statutory intervention, used when there are concerns for a child’s safety or well-being that meet the threshold for significant harm. Such a plan identifies what actions need to be undertaken, and remains in place until the risk is no longer present, or a CLA intervention is started.^7^

Current evidence shows children with a CLA intervention, and those known to Children’s Social Care, have significantly worse educational attainment than their peers, although there have been limited studies comparing these children with the general population.^8 9^ These children also have greater needs associated with their physical and mental health, and worse employment outcomes extending across the life course into adulthood.^10^,^12^

In the UK, financial pressures are mounting for local authorities, with many at risk of going bankrupt.^13^ Increases in Children’s Social Care costs are part of these pressures, with late intervention spending driving this while preventative and early intervention spending is falling.^14^ Understanding a child’s progression through the Children’s Social Care system after contact has been made will support the system to be responsive to demand.^15^ In 2017/2018, an analysis found that the average cost of a single CLA intervention was approximately £38 000 for that year, ranging up to £51 000.^16^

In local authorities of high deprivation rationing of services may be more acute.^17^ Evidence shows that deprivation and reduced preventative spending are significantly associated with worse OFSTED (Office for Standards in Education, Children’s Services and Skills) ratings,^18^ and ‘inadequate’ ratings are associated with an increase in Children’s Social Care interventions.^19^ Spending on Child in Need interventions and better OFSTED ratings are both shown to be associated with lower CLA rates.^20^

Our study applies an epidemiological and inequalities approach to the area of Children’s Social Care. Applying Rawls theory of justice to health, it follows that health inequalities are unfair and unjust and action on health inequalities should consider the social determinants of health.^21^ This approach has been applied to Children’s Social Care, and improvements to services must consider social determinants.^18^

Similar to the approach to tackling the social determinants of health to reduce health inequalities, our study aims to identify the risk profile to guide preventative interventions to reduce the numbers of higher cost, more intensive intervention (such as CLA).^17^ Preventative spending is associated with reduced intervention, however, deprived local authorities are more likely to use resources for interventions.^22^ The independent review in 2022 suggested moving the focus to supporting families,^23^and there are calls to use the principles of public health in this setting.^24^

The use of predictive modelling has been suggested as an option to identify children who may be at risk, however, there are concerns about the accuracy and ethical implications of such models.^25^ Some risk factors for becoming CLA have been identified. Maternal mental health and lifestyle during pregnancy, perinatal factors, household mental health problems and substance use are all predictors of a child becoming CLA.^26 27^

This study takes place in Liverpool, an area of high deprivation, which received a low OFSTED rating in 2023,^28^ and has among the highest rates of CLA in England.^29^ It is an area that may benefit from a more preventative approach given the evidence highlighting the effects of a poor OFSTED and high deprivation. Therefore, it is crucial to comprehend the trajectory within the system once a child is referred, and to better understand which children are most at risk to better target interventions to reduce that risk, and therefore cost. We use survival analysis to identify, among children known to social care in a deprived local authority, which demographic, household and service predictors were associated with becoming looked after. The next section will explain the methods we used, followed by the results and finally a discussion of how our findings may relate to policy, including a discussion of the strengths and limitations of our study.

## Materials and methods

### Study participants and data source

Our study used routinely collected, anonymised social care data for all children who received their first referral to Children’s Social Care team between 1 April 2019 and 8 March 2022. Data from the Children’s Social Care administrative system was linked with data on potential risk factors from other council departments, health services and police. This linkage occurred using a pseudonymised ID within the local authority. This linked data had already been received by the supporting families from separate sources and compiled into a linked database by the council to inform programmes supporting families with complex needs.

### Inclusion criteria

We included all children who became known to Children’s Social Care between 9 months before birth and the age of 18 inclusive (n=6380, with 307 outside this age range). We excluded those who had a Child in Need, Child Protection Plan or CLA intervention prior to their date of first referral to Children’s Social Care (n=66), as this suggests a referral prior to the date of first referral. Assessments that occurred before the date of the first referral were removed from our data as this temporal ordering of events was not feasible. We also excluded all unaccompanied asylum seeker children since their care status is unlikely to be related to risk factors of interest (n=203). The final cohort contained 5808 children. Finally, children estimated to be outside of the age range after a certain month had all subsequent months removed from the data.

### Outcome

Our outcome was the number of months between the first referral to Children’s Social Care and the start of the first CLA intervention. Children were censored when they reached March 2022, or became over 18 years old.

### Predictors

We included the following demographic measures:

- Age of the child at first referral—approximated as age by subtracting year of birth from year of first contact, categorised as pre-birth, less than 1-year old, between 1 and 4, between 5 and 9, between 10 and 14 and 15–18 years old.
- Ethnicity—available as recorded in the Children’s Social Care administrative system: white (white British, white Irish and other white), black (Caribbean, African, any other black), Asian (Bangladeshi, Chinese, Indian, Pakistani, any other Asian), mixed and other (Gypsy/Roma, Irish Traveller, other). Responses that were unknown or not stated were defined as missing.
- Sex of the child—categorised as male, female and missing. Responses that were unborn or indeterminate were defined as missing.
- Maternal age at birth—categorised as less than 18 years, 18–24, 25–34 and 35 years or older. Responses that were unknown or not stated were defined as missing.
- Index of Multiple Deprivation—a composite measure of deprivation available for small neighbourhoods (Lower Super Output Areas),^30^ categorised into five quintiles specific to Liverpool.

Other predictors were included separately from demographic factors and were mostly time-varying (other than Special Educational Needs (SEN)). Assessment data from the administrative system was time-varying, referring to an assessment of the child’s situation made by a social worker. The predictors were assumed to be constant in between assessments, and the period between referral and assessment used the exposure status from the first assessment. The other linked data covered a year period. These included:

- Drug and/or alcohol use—recorded at assessment in the Children’s Social Care administrative system split into present in the child and present in the household. This was time-varying.
- Domestic violence—either recorded at assessment or identified from linked police data indicating a perpetrator or victim in the household. This was time-varying.
- Mental ill health—either recorded at assessment or identified from linked health data, split into present in the child and present in the household. This was time-varying.
- Physical health conditions—split into present in the child and present in the household, recoded at assessment. This was time-varying.
- Learning disability—split into present in the child and present in the household, recoded at assessment. This was time-varying.
- Neglect and abuse (emotional, physical, sexual)—recoded at assessment. This was time-varying.
- SEN—in the child, recorded in the master data.

The use of other statutory interventions (Child in Need or Child Protection Plan) after the initial assessment was dichotomised as either used one or more times during the study period, or not used at all.

### Statistical analysis

**Figure 1:**
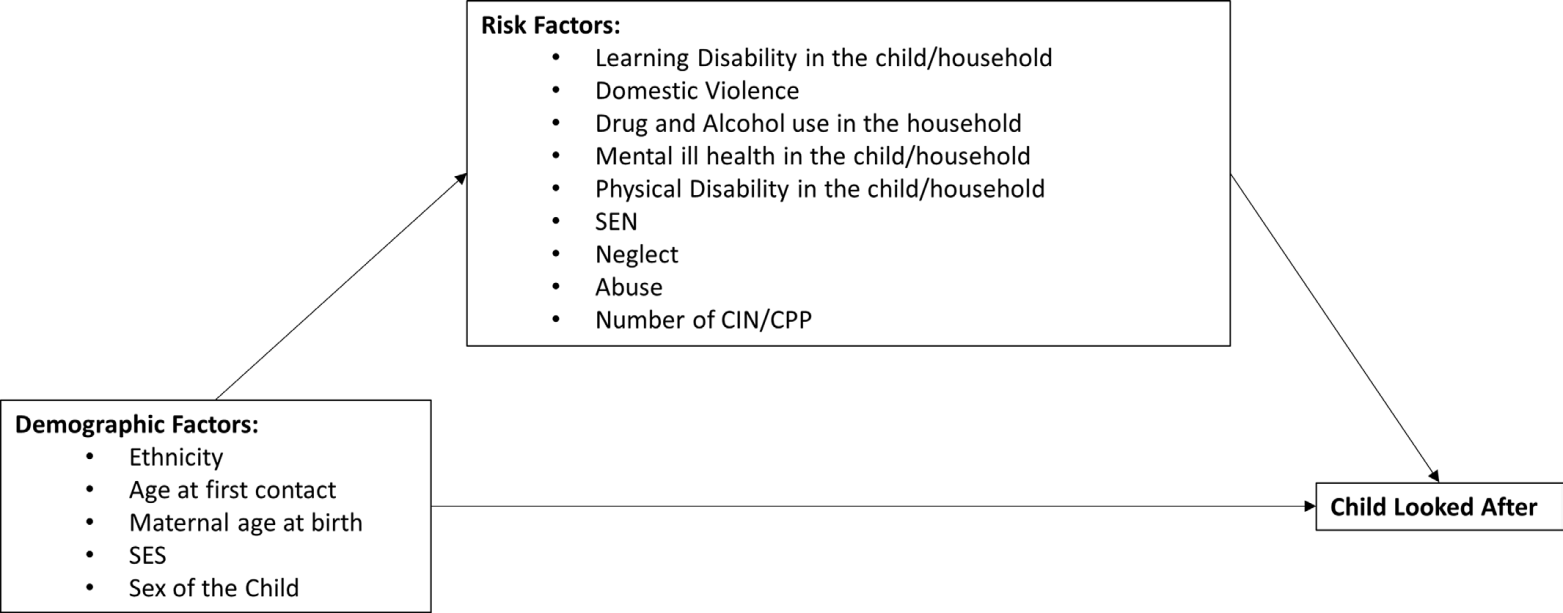
Logic model that underpins our analysis approach, showing the association between demographic factors and becoming a child looked after are potentially explained by more proximal risk factors. CIN, Child in Need intervention; CPP, Child Protection Plan; SEN, special educational needs; SES, socioeconomic status.

Figure 1 shows the simplified theoretical pathway that informs our analysis, based on the variables available in the data. We assume that potential differences seen in the risk of becoming CLA according to demographic factors are potentially explained (or mediated) by other more proximal risk factors.

First, we produced a descriptive analysis of the children who became known to social care during the study period, including demographic factors, and whether a Child in Need or Child Protection Plan intervention had been used before the outcome, between children who became looked after and children who did not. Second, we produced a descriptive analysis of the risk factors. As many of these risk factors were time-varying, we presented these as proportions of those present when the CLA occurs.

Then to explore the association between baseline demographic factors and the risk of being taken into care we plot survival curves stratified by these factors, indicating factors that are associated with the risk of children being taken into care. To assess the independent effect of demographic and other risk factors we used a discrete time hazard regression model. This used monthly data on each child (the person-period interval) and our outcome of interest—either first start a CLA intervention or become censored by reaching the end point of the data (March 2022) or reaching a estimated age over 18. We modelled this as our baseline hazard as the number of months from referral to Children’s Social Care. The functional from of the baseline form was assessed visually (online supplemental figure 1). As the functional form was approximately linear, we included time to event as a linear term in the model. We use a complementary log–log link function in the regression model—which is analogous to a continuous time Cox proportional hazard model, in a discrete time set-up. This means that exponentiated coefficients form the model can be interpreted as HRs.^31^,^33^ See model description in online supplemental material for a more detailed description.

We estimated two models; a demographic-only model, which includes the demographic factors and a model that includes demographic factors, other predictors and whether another statutory intervention (Child in Need or Child Protection Plan) was used before the outcome (either becoming CLA or censoring). We have included two models as we are interested in how the relationship between demographics and CLA change when risk factors are accounted for—given that demographic factors are unchangeable and other risks are potentially amenable to intervention, understanding how the effect of demographics can potentially be explained by other risks can be helpful when predicting who is at risk.

We ran a sensitivity analysis showing the HR’s when the time to event is included as a categorical variable. Missing data were removed via listwise deletion. All analyses were conducted in R statistical software V.4.3.0.^34^

### Ethical approval

Not required as this was a secondary analysis of anonymised data, and data sharing was agreed with Liverpool City Council Children’s Social Care team.

### Patient and public involvement

Our study is part of the RESTORE (Research for Equitable SySTem RespOnse and REcovery) programme of work. This programme involved engagement sessions with the public which has informed our topic of research and the priority areas. The results from this work will be used to feed into our ongoing engagement work with young people, families and Children’s Social Care about focusing on their priorities.

## Results

### Descriptives

Overall, 5808 children were referred to Children’s Social Care between 1 April 2019 and 8 March 2022, 377 of whom became CLA, covering a total of 117 699 months between referral and either outcome or censoring. Table 1 shows the characteristics of the children referred sample. A high proportion of children referred come from the most deprived neighbourhoods of Liverpool and a relatively high proportion of children are from ethnic minority groups, 32% (excluding missing). This, for example, compares to 22% of under 25 years old in Liverpool being of ethnic minority groups in the 2021 census. The more frequent age group to be referred was 1–4 years old, the most common maternal age group was 25–34 years old, and there were a similar number of girls and boys referred.

**Table 1:** The percentage of children who became looked after by demographic factors and whether the child had a CLA intervention or not

	Total sample number	Percentage who became CLA
Local IMD quintile		
1 - least deprived	343	5.2
2	637	6.4
3	864	4.1
4	1433	4.3
5 - most deprived	1966	5.6
Missing	565	19.6
Ethnicity		
White	3607	6.2
Asian	394	7.6
Black	439	6.8
Mixed	397	8.1
Other	486	7.4
Missing	485	4.9
Age at first referral		
Pre birth	379	16.9
<1	924	14.9
1–4	1421	3.7
5–9	1242	2.8
10–14	1160	2.3
≥15	682	8.9
Maternal Age at Birth		
<18	155	7.1
18–24	1877	6.1
25–34	2669	5.9
35+	712	6.0
Missing	395	12.9
Sex of the Child		
Female	2742	5.8
Male	2792	7.8
Missing	274	0.0
CIN		
No	4627	7.2
Yes	1181	3.7
CPP		
No	5072	3.5
Yes	736	26.9
Assessed		
Yes	5191	6.8
No	617	4.1

CINChild in NeedCLAchild looked afterCPPChild Protection PlanIMDIndex of Multiple Deprivation

Online supplemental figure 2 shows the survival curves indicating the proportion of children that remained out of care (ie, not CLA) over time. For age at referral, children who become known to services earlier were more likely to become looked after particularly in the first 10 months after initial referral. Boys were more likely to become looked after than girls. Children from ethnic minority groups were also more likely to be taken into care than children of white ethnicity. There were no large differences in the proportion of children referred to Children’s Social Care who became CLA by the level of deprivation and by maternal age. Children who had a Child in Need intervention prior to the endpoint were less likely to become CLA while predictably, given its use in situations where children are deemed at risk of significant harm, children who had a Child Protection Plan intervention prior to the endpoint were more likely to become CLA.

**Figure 2:**
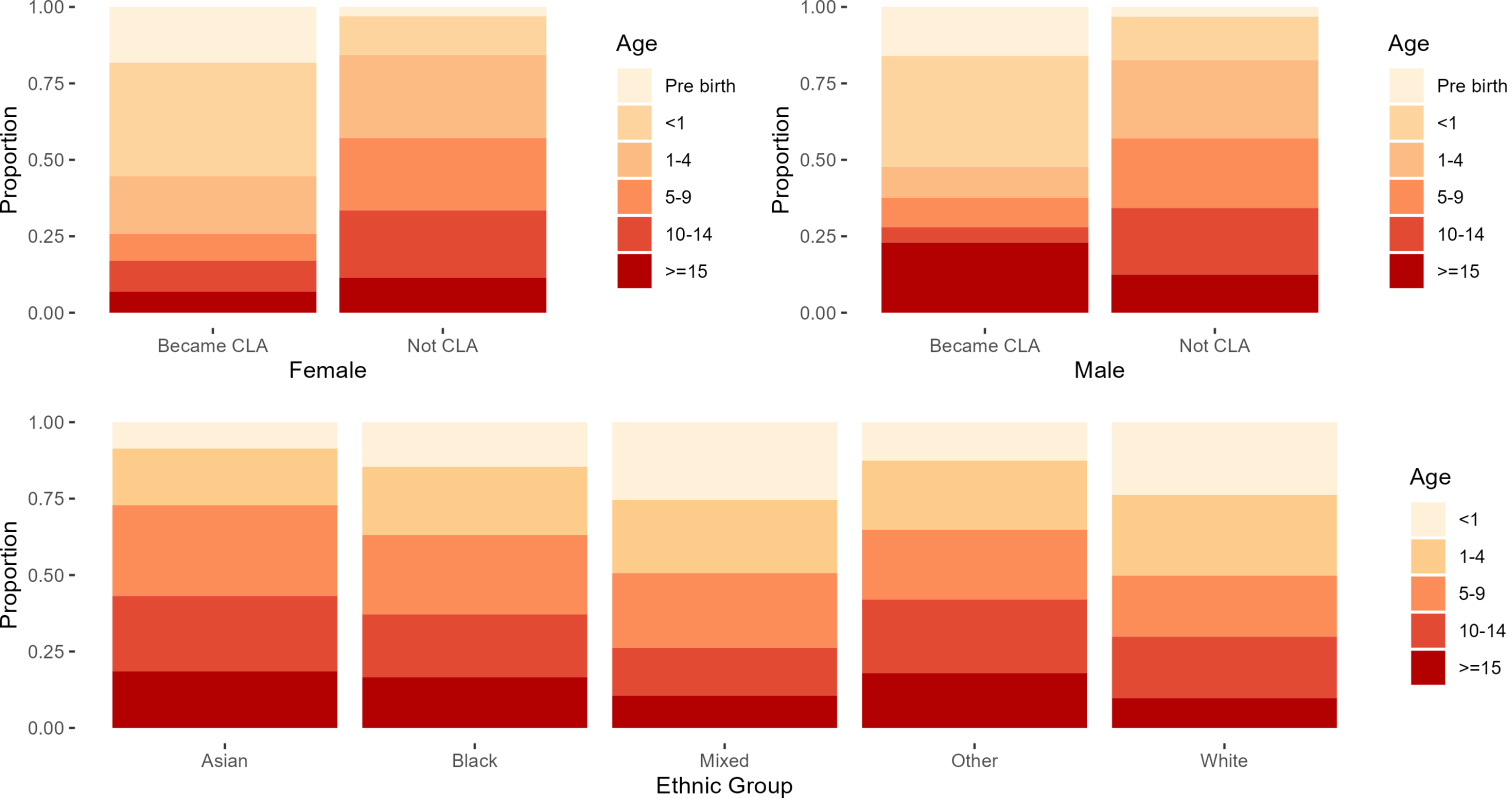
Scarf plots showing—left panel: distribution of age at first referral by child looked after (CLA) status, split by sex. Right panel: distribution of age at first referral by ethnicity of the child.

Figure 2 shows the pattern of age at first contact by sex and ethnicity. Boys who become known to services at 15 or older are more likely to become looked after, but this is not the case for girls. For children who are white or of mixed ethnicity, approximately a quarter became known at less than 1 years old or pre-birth. For Asian ethnicity, less than 10% were in this age group, while for black and other ethnicities, it was less than 15%. Conversely, less than 30% of children who are white or of mixed ethnicity became known to Children’s Social Care older than 10, compared with over 40% of Asian children, and 37% of black children. Among the children who had a CLA intervention, over half were exposed to poor household mental health, drug and/or alcohol abuse in the household and neglect. A full list of these is included in the supplementary material (online supplemental table 1).

### Discrete time model

Table 2 shows the HRs from the discrete time hazard model including demographic factors only. When compared with children aged 1–4 at first referral, those who were known pre-birth or in the first year were more likely to become looked after. When controlling for other demographic factors children referred from the most deprived quintile of lower super output areas were 2.45 times more likely to become CLA than those referred from the least deprived quintile. This finding is different from our descriptive analysis (table 1), demonstrating that other demographic variables effect this association. Referred children born to mothers aged between 25 and 34 were significantly less likely to become CLA than those born to 18–24 years old mothers. While black, Asian and mixed children had a greater risk of becoming CLA in this analysis this was not statistically significant at the 5% level.

**Table 2:** Discrete time model output for hazard of becoming child looked after. Model includes demographic factors only

	HR	LCL	UCL
Local IMD quintile			
1 - least deprived	Ref	.	.
2	1.67	0.62	4.48
3	1.83	0.71	4.72
4	2.01	0.81	5.03
5 - most deprived	2.47	1.00	6.08
Ethnicity			
White	Ref	.	.
Asian	1.26	0.69	2.30
Black	1.20	0.73	1.98
Mixed	1.20	0.74	1.94
Other	0.88	0.50	1.56
Age of child at first contact			
Pre birth	5.02	3.06	8.25
<1	2.98	2.08	4.27
1–4	Ref	.	.
5–9	0.75	0.48	1.16
10–14	0.56	0.34	0.92
≥15	0.60	0.32	1.13
Maternal age at birth			
<18	1.02	0.47	2.2#2
18–24	Ref	.	.
25–34	0.72	0.53	0.97
35+	0.81	0.52	1.24
Sex of child			
Female	Ref	.	.
Male	1.02	0.78	1.33

Model includes demographic factors only*.*

IMDIndex of Multiple DeprivationLCLLower 95% Confidence LimitUCLUpper 95% Confidence Limit

**Figure 3:**
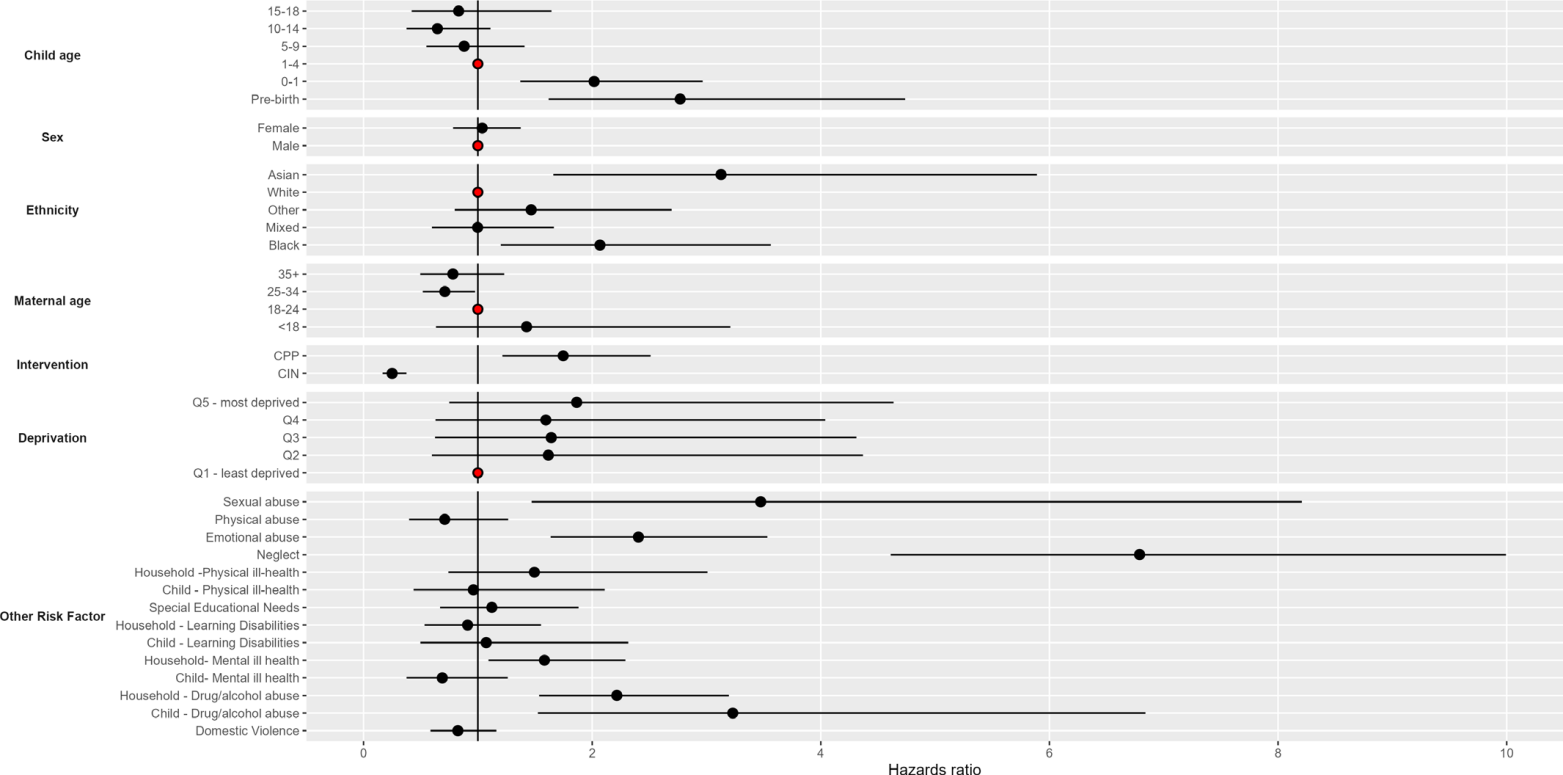
Hazard ratios from discrete time hazard model, showing increased risk of becoming a child looked after. Model includes demographic factors, statutory interventions and risk factors, CIN, Child in Need; CPP, Child Protection Plan. (Red markers indicate reference categories).

Figure 3 shows the HRs from the model including for both demographic, other interventions and other risk factors. Unlike the demographic model, when we control for potential mediators, Asian and black children referred to Children’s Social Care had a significantly higher hazard of CLA, compared with referred white children (HR 3.13 95% CI 1.66 to 5.89 for Asian children, HR 2.07, 95% CI 1.20 to 3.56 for black children). The effect of being referred at under 1 or pre-birth was attenuated (HRs 2.02 and 2.77, respectively), however, the HR remained significant. Referred children born to mothers aged between 25 and 34 remained significantly less likely to become CLA than those born to 18–24 years old mothers (HR 0.71, 95% CI 0.52 to 0.98).

Children who had a Child in Need intervention, independent of the other predictors, were over four times less likely to have a CLA intervention (HR 0.25, 95% CI 0.17 to 0.38), while those with a Child Protection Plan intervention were significantly more likely to become CLA (HR 1.75, 95% CI 1.21 to 2.51). When compared with referred children not exposed, those exposed to mental ill health in the household were more likely to become looked after (HR 1.58, 95% CI 1.09 to 2.29). When compared with referred children not exposed, those exposed to drug and/or alcohol use in the household, were also more likely to become looked after (HR 2.22, 95% CI 1.54 to 3.20).

Children with mental ill health did not have an increased hazard of becoming looked after, while those assessed as using drugs and/or alcohol were more likely to become looked after (HR 3.23, 95% CI 1.53 to 6.84). When compared with children not assessed as exposed to neglect, those who were assessed as exposed to neglect were more likely to become looked after (HR 6.79, 95% CI 4.61 to 9.99). When compared with children not assessed as exposed to emotional or sexual abuse, those assessed as exposed were more likely to become looked after. This model was run with time to event included as a categorical term rather than a linear term; no substantial changes were noted (online supplemental table 2).

## Discussion

### Key findings

Using routinely collected data from a local authority Children’s Social Care team, we identified the risk factors that were associated with becoming CLA among children referred to Children’s Social Care. Of the 5808 children included, 377 became looked after. The key factors that were associated with increased hazard were neglect, sexual abuse, drug and/or alcohol use in the household or in the child, mental ill health in the household, emotional abuse and Child Protection Plan intervention. The risk factor associated with the largest increase in hazard was neglect, which was associated with a nearly seven times increase compared with those not assessed as exposed to neglect. Child in Need intervention significantly decreased hazard of becoming CLA.

Regarding demographics, earlier age of referral to Children’s Social Care was associated with significantly increased hazard in both models. Notably, ethnicity only became a significant factor when risk factors were also included. Black and Asian children both had a significantly higher hazard of becoming CLA than white children. Black and Asian children were referred to Children’s Social Care at older ages than white children. Increased hazard of CLA was seen as deprivation increased, however, this was only significant for the most deprived children in the demographics-only model.

When considering the potential mediation of demographic factors through risk factors the findings are that risk factors mediate the effects of deprivation, attenuating the hazard of higher deprivation on CLA. Conversely, risk factors mediate the effects of ethnicity in the opposite direction—inclusion of risk factors result in increased ethnic variation of children becoming looked after among children already referred to Children’s Social Care.

### Relevance to other studies

Our findings that mental ill health, and drug and alcohol abuse in the household are both significant risk factors for a CLA intervention are in line with the existing evidence base. A register-based study from Denmark found depression, bipolar disorder and schizophrenia all increased risk of CLA.^35^ A population-based study from Canada found that mental health and drug use were both associated with CLA.^27^ Maternal alcohol use has been shown to increase the risk of contact with Children’s Social Care in Australia.^36^ These findings are partially in line with the class analysis by Hood *et al*.^37^ Parental mental health, and drug and alcohol abuse overlapped as a category of demand in Children’s Social Care, however, our results suggest these are risks independent of each other.

Our finding that the presence of domestic violence is not associated with increased risk of CLA in Liverpool somewhat conflicts with existing evidence. A latent class analysis found that domestic violence exposure was a key factor in referral and demand, however, domestic violence alone was in a low proportion of CLA interventions.^38^ Additionally, our findings support the idea that the combined influence of poor mental health, drug use and domestic violence is not well evidenced and the prioritisation of all three is not indicated.^39^

Evidence from the USA showed that parents with learning disabilities were more likely to be known to Children’s Social Care,^40^ however our results suggest that once known to services, the presence of learning disabilities in the household does not increase the risk of a CLA intervention.

Our results for ethnicity are notable—an independent review for the Department for Education found that in England, white and mixed ethnicity children had higher rates of CLA intervention within a year of referral to Children’s Social Care.^41^ A 2017 study found that black children were over-represented in out-of-home CLA rates when compared with white children. Conversely, Asian children were comparatively under-represented.^42^ A systematic review also found persistent over-representation of black children, suggesting racial discrimination was a major contributor to this.^43^

Our findings show that the risk of CLA intervention is higher for black and Asian children compared with white children only when risk factors are adjusted for. A 2020 analysis found significant differences in intervention rates by ethnic group, however this was complicated by deprivation.^44^ The socioeconomic gradient was weaker in black and Asian children than in white children; the high levels of deprivation in Liverpool may explain why these inequalities are not seen in the initial model. Evidence has shown that risks outside of the home are more prevalent among black and Asian children who become CLA; these risks are not captured within our model.^45^ Our findings demonstrate that this relationship is influenced by other factors, and further disentangling is needed to better understand the contributions of ethnic bias (both in society and services) and the role of risks and needs.

The finding that a Child in Need intervention reduces hazard may suggest that Child in Need interventions are being used early and are effective at reducing future need for more acute intervention. Evidence suggests early interventions are effective at preventing harm and subsequently the need for CLA.^46^ Conversely, this may suggest risk assessment practices are working as intended.

Our findings in relation to deprivation are in keeping with existing evidence; living in more deprived quintiles is more common among the children who are referred to Children’s Social Care, or become CLA, than in the Liverpool population. These findings reflect the national picture.^47^ Our findings for the impact of deprivation on CLA specifically are smaller than other studies,^26^ however this is likely due to our comparison group being children already known to Children’s Social Care rather than the whole population.

### Strengths and limitations

Our study has several strengths. First, by using both administrative and linked data, we can include a rich variety of variables into one model. This allows us to adjust for a range of risks and demographic variables, and better understand the significance of such risks to a CLA intervention. Uniquely, we include other statutory interventions (Child in Need and Child Protection Plan) in our analysis.

Second, by using routine Children’s Social Care data, our data are representative of children who have been referred. Although our results should not be interpreted as causal, due to missing variables and potential biases, our results can help risk prediction. Finally, as we are only looking at children who have been referred, our results have a clear importance to those who work in a Children’s Social Care setting.

A number of limitations should be considered when interpreting our results. First, as our comparison group is children referred to Children’s Social Care that have not received a CLA intervention, we must be cautious in generalising our results to the total population. This is particularly relevant as our sample skews towards more deprived than the general Liverpool population. Selection bias will be introduced; for example, children exposed to risk factors that increase the likelihood of a CLA intervention may also increase the likelihood of referral to Children’s Social Care .

Second, the generalisability of our findings to other settings internationally is influenced by the variations in Children’s Social Care systems. Meanwhile, the generalisability to other UK settings is affected by the Liverpool context; for example, the high levels of deprivation and resource constraints may lead to reduced intervention relative to less deprived local authorities.^17^

Third, the frequency of assessments in our sample may introduce measurement bias. We have assumed the factors that are highlighted at assessment are unchanged until a child is reassessed. This is potentially less accurate the further away from the assessment the child moves, and children with greater needs may be more likely to be reassessed.

Fourth, other reasons to be censored, such as moving out of the area or dying are not captured in our data. Fifth, other risk factors were unavailable for many of our cohort. Data on police contacts, educational data and more detailed data on health (such as healthcare use and specific diagnoses) are all potential risk factors for CLA intervention that have not been included in our analysis. Sixth, although small numbers, if missing data were unevenly distributed this may potentially bias results.

Finally, our results focus on risk prediction and our analysis is associational. Thus, causal interpretations of our findings should not be made. Our findings can be used to highlight which population groups could be targeted for preventative interventions and some of our findings merit further investigation (eg, identify wider determinants for Children’s Social Care interventions). Our approach to risk prediction will be replicable in other areas using routine data.

### Policy implications

Our results have some key implications for policy. The ‘What works centre for Children’s Social Care’ has a number of suggestions, including system change, family skills training and interventions to influence family finances.^48^ Changes to implement universal policies that improve household mental health and reduce drug and alcohol use may be particularly useful. Mental health comorbidity has been shown to increase the risk of CLA intervention in mothers with drug and alcohol use.^49^

Our ethnicity findings may suggest the role of risks are different in groups, and that risks for a CLA intervention for ethnic groups are not captured in our data. Considering older age at referral for both black and Asian children, this suggests that the risk of harm for these children may be identified late. There is a need for research to better understand the aetiology of harm experienced by children of different ethnicities, so that appropriate preventative approaches can be developed. Conversely, the risk profile for these groups may vary, for example, structural, contextual and community risks may be of more importance than home risks for different ethnic groups.

The relationship between deprivation and both referral to Children’s Social Care and CLA highlights the potential for social care to reduce inequality through action on the stresses associated with poverty and the need for services to act in a ‘Poverty-Aware’ way.^50^ Abuse and neglect are key drivers of a CLA intervention. This is supported by our evidence and interventions to reduce these is indicated.

Finally, the relationship between age at referral and CLA intervention should be considered. Children who are referred before their first birthday are at increased risk of CLA. Part of this group is potentially the children born into care, who require specific support such as specialist multidisciplinary support for parents and social worker teams dedicated to the pre-birth period.^51^ Children who are referred this early should be given assessment and intervention that better meets their specific needs incorporating the concerns and experiences of parents.^52^ Boys aged 15 years old and over appeared disproportionately likely to experience a CLA intervention, considering the proportion of boys who were referred. Evidence shows a relationship between cuts to preventative services and older children entering care.^53^ This requires further investigation as this is a group who are potentially at increased risk of poor outcomes and instability, with high resource pressures.

Such a preventative approach would follow the proportionate universalist approach, moving away from tiered service and improving access to specialist expertise,^15^ rather than waiting for more intensive needs prior to intervention and fit with the family-focused approach.^23^

## Conclusion

Our study of routinely collected Children’s Social Care data found that the key factors that increased the hazard of a CLA intervention were neglect, sexual abuse, drug and/or alcohol use in the household or the child, mental ill health in the household, emotional abuse and Child Protection Plan intervention. Ethnicity increased hazard only when other risk factors were included, while having a Child in Need intervention significantly decreased hazard of becoming CLA. Through applying a public health lens to the area of Children’s Social Care, we identify the profile for children who are most at risk of a CLA intervention. Such an approach can be used to identify population segments for early intervention.

## supplementary material

10.1136/bmjph-2024-001130 online supplemental file 1

## Data Availability

Data may be obtained from a third party and are not publicly available.
