# Supplementary material for: What factors are associated with children being taken into care by the state after initial contact with services? A survival analysis of Children’s Social Care data in Liverpool
Source: BMJ Public Health. 2024 Oct 7;2(2):e001130. doi: 10.1136/bmjph-2024-001130 (PMC11816848; doi:10.1136/bmjph-2024-001130)
Supplement: online supplemental file 1 [file bmjph-2-2-s001.pdf]

Figure S1: Baseline hazard for the number of months from referral to complementary log-log link function for CLA

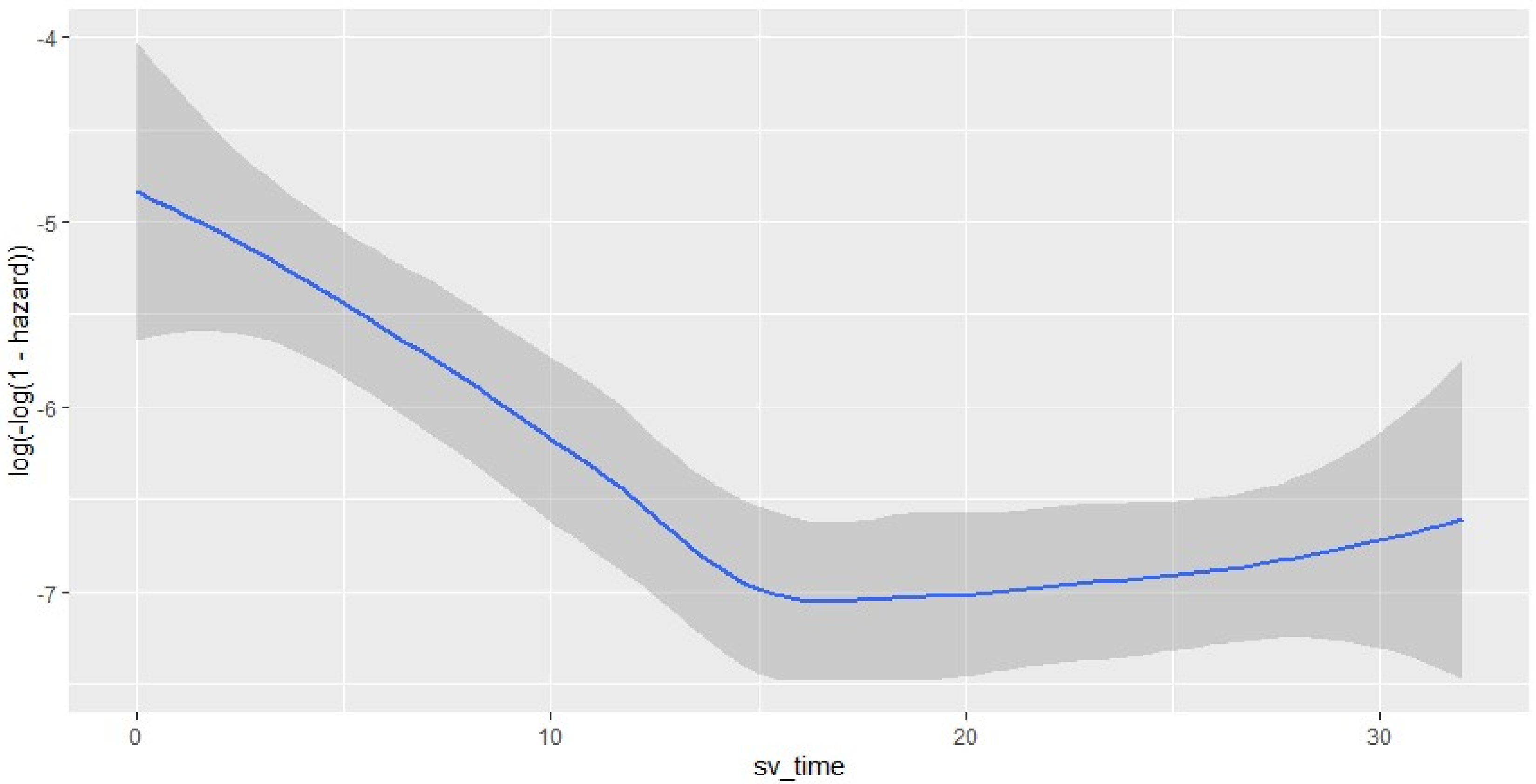

Model Description

In our main analysis, our primary outcome of interest is the time from first referral to CLA, therefore we constructed a survival model. We modelled the time from first referral to CSC to becoming CLA through a discrete time model with a complementary log-log link function:

$$log\left(-log(1-\text{hazard})\right)$$

The level of observation was month per individual (the person-period interval). In our model the equation is:

$$y_{it} = Demographic\ Predictors_{it} + Other\ Predictors_{it} + Statutory\ Interventions_{it} + Time_t + \varepsilon_{it}$$

y_{it} is a dichotomous variable which takes the value 1 if child *i* became CLA in month t, or 0 if child *i* did not become CLA in month t.

$Demographic\ Predictors_{it}$ are the demographic variables recorded for child *i* during month t. These variables are age at referral, sex, deprivation and ethnicity. These variables are time invariant so do not change according to month t.

$Other\ Predictors_{it}$ are drug and/or alcohol use (child or household), domestic violence, mental ill health (child or household), physical health conditions (child or household), learning disability (child or household), neglect and abuse (emotional, physical, sexual), or Special Educational Needs. These are dichotomous variables which take the value 1 if child *i* is exposed to these variables in month t, or 0 if not. Special Educational Needs is time invariant.

$Statutory\ Interventions_{it}$ are dichotomous variables which take the value 1 if child *i* has had a Child in Need or Child Protection Plan during the study period, or 0 if not. These variables are time invariant so do not change according to month t.

$Time_t$ is a continuous variable for the number of months between referral to Children's Social Care and time t.

ε_{it} is the error term.

Table S1: Percentage prevalence of risk factors in month when CLA intervention starts.

Risk Factor		%	Number Missing
Exposed to Domestic Violence	Yes	48.9	25
Drug and Alcohol use in the Household	Child	2.8	25
	Household	53.4	25
Mental Ill Health	Child	5.4	25
	Household	58.4	24
Learning Disabilities	Child	2.8	25
	Household	11.6	25
Physical Health Conditions	Child	2.3	25
	Household	6.0	25
Special Educational Needs	Yes	6.1	0
Neglect	Yes	52.6	25
Abuse	Emotional	44.3	25
	Physical	7.1	25
	Sexual	2.8	25

Figure S2. Survival Curves showing the remaining proportion of children not taken into care, stratified by age group, sex, ethnicity, deprivation, maternal age, and whether the child received a child protection plan or child in need intervention. Black, Asian, mixed and other ethnic groups were grouped into this one category for ease of visualisation.

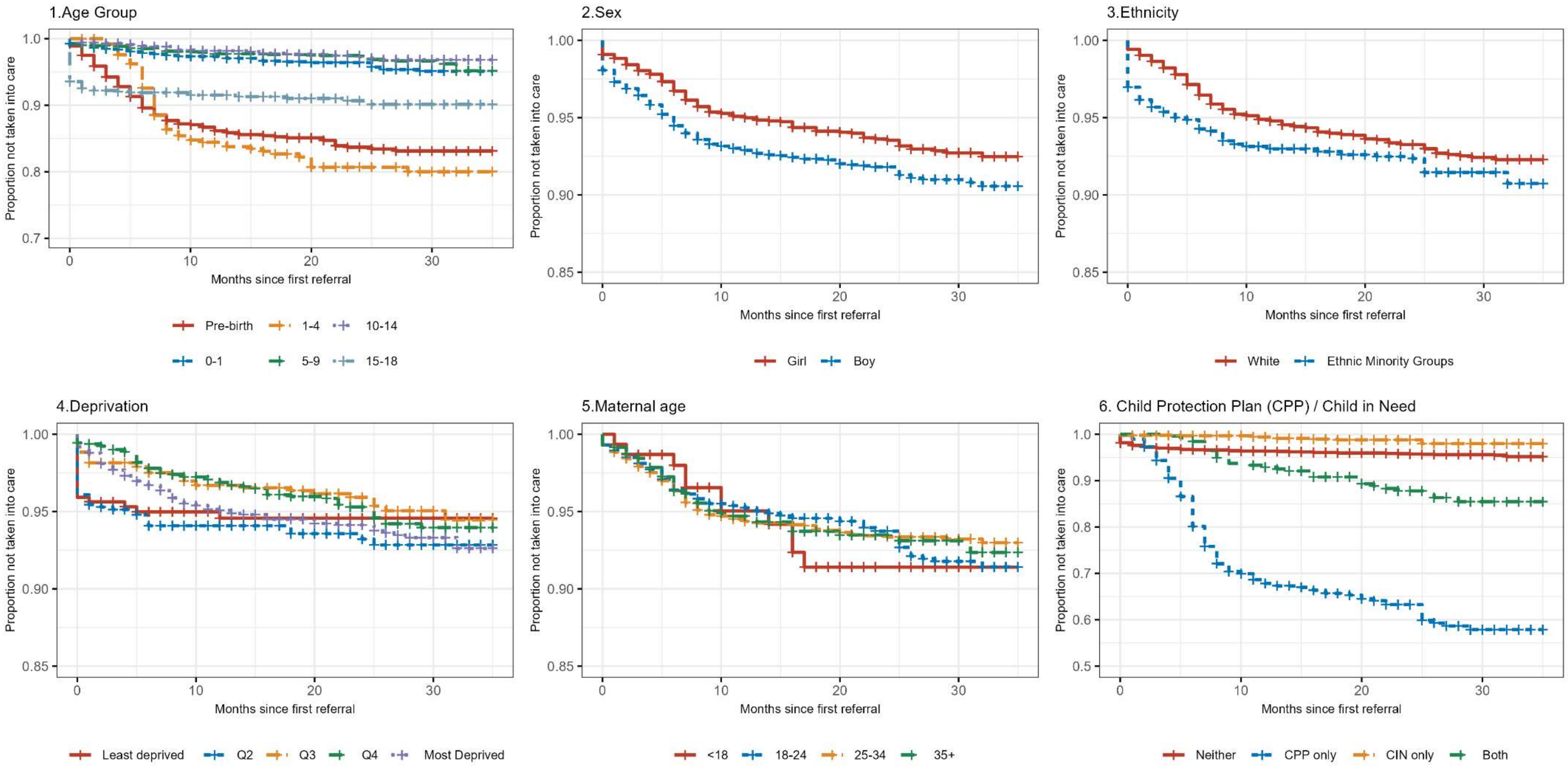

Table S2: Discrete time model output for hazard of becoming child looked after when time to event is included as a categorical variable. Model includes demographic factors, statutory interventions, and risk factors. IMD: Index of Multiple Deprivation, CIN: child in need, CPP: child protection plan

		HR	LCL	UCL
Local IMD Quintile	1 - least deprived	REF	.	.
	2	1.57	0.58	4.23
	3	1.60	0.61	4.20
	4	1.54	0.61	3.90
	5 - most deprived	1.81	0.73	4.51
Ethnicity	White	REF	.	.
	Asian	3.05	1.62	5.74
	Black	2.05	1.19	3.54
	Mixed	0.99	0.60	1.66
	Other	1.37	0.74	2.55
Age at first referral	Pre birth	3.16	1.61	4.73
	<1	2.16	1.36	2.96
	1-4	REF	.	.
	5-9	0.91	0.56	1.42
	10-14	0.70	0.38	1.12
	>=15	0.95	0.43	1.68
Maternal Age at Birth	<18	1.49	0.66	3.38
	18-24	REF	.	.
	25-34	0.70	0.51	0.97
	35+	0.78	0.50	1.23
Gender	Female	REF	.	.
	Male	1.04	0.78	1.38
CIN	No	REF	.	.
	Yes	0.25	0.17	0.38
CPP	No	REF	.	.
	Yes	1.77	1.23	2.54
Exposed to Domestic Violence	No	REF	.	.
	Yes	0.83	0.59	1.17
Drug and Alcohol use in the Household	Child	2.94	1.36	6.36
	Household	2.21	1.53	3.19
Mental Ill Health	Child	0.66	0.36	1.22
	Household	1.56	1.08	2.26
Learning Disabilities	Child	1.08	0.50	2.33
	Household	0.90	0.53	1.54
Physical Health Conditions	Child	1.01	0.46	2.22
	Household	1.49	0.74	3.00
Special Educational Needs	No	REF	.	.
	Yes	1.11	0.66	1.86
Neglect	No	REF	.	.
	Yes	6.73	4.57	9.92
Abuse	Emotional	2.40	1.63	3.53

	Physical	0.71	0.40	1.26
	Sexual	3.45	1.46	8.12
